# Complication profile of two surgical techniques, neurosurgical experience, and operative setting for pediatric external ventricular drainage implantation

**DOI:** 10.1007/s00381-026-07413-7

**Published:** 2026-08-12

**Authors:** Tommaso Araceli, Sarah Klarner, Florian Zeman, Amer Haj, Sebastian Siller, Martin Proescholdt, Nils Ole Schmidt, Christian Ott

**Affiliations:** 1 Department of Neurosurgery, University Regensburg Medical Center, Regensburg, Germany; 2 Center for Statistics and Clinical Studies, University Regensburg Medical Center, Regensburg, Germany

**Keywords:** Ventricular drain, Hydrocephalus, Bolt kit-screw, Surgical experience, Complications

## Abstract

**Purpose:**

External ventricular drain (EVD) insertion, a standard procedure for treating acute hydrocephalus, is also associated with significant risks. This retrospective study compares two surgical techniques routinely employed at our clinic and analyzes the influence of surgeon experience and the operative setting on complication rates in a pediatric population.

**Methods:**

The study population was divided into two cohorts based on the surgical procedures used to access the ventricular system: the BoltKit system (BS) or drain tunnelling (DT). Statistical analysis balanced age, sex, site and side of the drainage, neurosurgical experience, i.e. junior surgeons (JS) or senior surgeons (SS), and operating settings, i.e. intensive care unit (ICU) or operating room (OP), with complications such as cerebrospinal fluid infection, multiple insertion attempts (MIA), malpositioning, revision, and bleeding.

**Results:**

This retrospective study included 101 patients (62 males, 39 females, mean age 7.1 years): 79 were treated with DT, and 22 with BS. Overall, 81 EVDs were placed frontal, 18 occipital, 68 at the right and 25 at the left side; 22 revisions (22%), 13 infections (13%), 11 MIA (11%), 10 malpositionings (10%), and 4 bleedings (4%) occurred. MIA, malpositionings, and revisions were significantly more frequent after BS than DT (p = 0.012, p = 0.038, p = 0.020). Revisions were also more common in ICU than OP insertions (p = 0.002) and in JS vs. SS (p = 0.018). Infections were significantly associated with frontal catheter placement (p = 0.018) but not with technique. In multivariable models, both DT and SS reduced the risk of MIA independently, while an ICU setting predicted the need for revision independently.

**Conclusion:**

In children, DT is associated with fewer complications than BS. Both surgical experience and the operative setting significantly influence outcome, highlighting the importance of careful technique selection and supervision in pediatric EVD placement.

## Introduction

Hydrocephalus is a common disorder of the physiology of cerebral spinal fluid (CSF) that results in the abnormal expansion of the cerebral ventricles [1]. Hydrocephalus can result from many underlying conditions that disrupt the circulation of CSF [2]. Infants commonly present with progressive macrocephaly whereas children older than 2 years generally present with signs and symptoms of intracranial hypertension [1]. More than 60% of children with hydrocephalus require their first surgical treatment during infancy [3]. The relationship between the presence of hydrocephalus and poor outcome has been clearly demonstrated [4]; thus, acute hydrocephalus is a key therapeutic target during the acute phase to improve neurological outcome [5]. During the acute phase, temporary treatment options include CSF puncture and use of ventricular access devices and ventricular subgaleal shunts. On of the most frequently performed neurosurgical procedures is the placement of an external ventricular drain (EVD) [3, 6]. Although EVD placement is a commonly performed procedure, it is associated with considerable risks, including misplacement, bleeding, dislodgement, and CSF infection [7, 8]. Studies conducted on adults and in general populations have shown that inserting an EVD through a bolt at the bedside is safer than the standard tunneled EVD insertion technique used in operating theaters. Bedside insertions have shown a significantly higher chance of optimal placement and a significantly lower chance of accidental discontinuation or complications such as reinsertion [7, 9–12]. To our knowledge, no comparative analysis has yet been conducted exclusively on the pediatric population. Therefore, this study compares the complication rates of two surgical techniques that are routinely performed at our clinic.

## Materials and methods

### Study design and ethical approval

This single-center, retrospective study included all children with acute hydrocephalus who underwent an EVD insertion at our clinic between January 2018 and January 2024. Patients older than 18 years of age and patients with missing pre- or postoperative clinical and radiological data or missing follow-up data were excluded. All cases were discussed by our internal neurosurgical case conference, and the decision to operate was made due to the presence of symptomatic hydrocephalus. Ethical approval from the Regensburg University Institutional Ethics Review Board (vote no. 26–4567-104) was obtained in accordance with German ethical and regulatory standards and the Declaration of Helsinki (7th revision, 2013). The data protection concept, established according to the national legislation, was strictly followed.

### Data collection and definitions

The following variables were collected from the electronic patient files of the SAP® software (SAP® Deutschland SE & Co.KG, Walldorf, Germany) and the radiological and medical reports: age, sex, site and side of the EVD, complications such as revisions, multiple insertion attempts (MIA), malpositioning, bleedings, and infections, surgical techniques, surgeon experience, and the operative setting. Variables were defined according to the literature as follows. Revision was defined as any surgical reintervention required due to malfunction, displacement, obstruction, or complication of the EVD after the initial insertion, leading to symptomatic insufficiency. MIA referred to more than one ventricular punction necessary to achieve successful catheter placement during the first procedure. Malposition was defined as radiologically confirmed placement of the catheter tip outside of the ventricular system, such as in intraparenchymal and subdural locations. Bleeding denoted procedure-related intracranial hemorrhage (ventricular, parenchymal, or subdural) detected on postoperative imaging. Infection was considered present in cases of culture-proven CSF infection or ventriculitis occurring while the EVD remained in situ, with corresponding clinical or laboratory evidence of infection. For the comparative analysis of infections, 14 patients were excluded due to either a history of previous intracranial or systemic infections or insufficient data to define infection according to the study criteria. The operative setting was recorded as either an operation room (OP) or an intensive care unit (ICU).

### Surgical techniques and study groups

Our clinic adheres to internationally accepted standards for the site of EVD insertion. The catheter is inserted into the anterior horn of the lateral ventricle through a burr hole located just anterior to the coronal suture, along the midpupillary line. Catheter placement was individualized according to patient-specific anatomy, resulting in a dorsal positioning depth ranging approximately 9–11 cm above the nasion, on the midline, and 2,5 cm lateral to it. External landmarks such as the inner canthus and the external auditory meatus [13] are used to guide the insertion. Patients were dichotomized according to the surgical procedure used to access the ventricular system: 1) use of an electric or mechanical compressed-air drill, insertion of the EVD, and under-scalp drain tunnelling (DT) with a minimum length of 5 cm according to established protocols described in the literature [14], or 2) use of an electrically or manually operated twist trepanation, insertion of the EVD, and fixation with a skull-bolted screw (BS, Raumedic AG, Germany). In both techniques, the catheter was additionally fixed to the scalp. The BS technique was not applied to children younger than 3 years. In accordance with institutional standards, all patients received an antibiotic-impregnated catheter.

### Surgeon experience

Among the variables collected, we examined the level of experience of the operating surgeon and stratified cases into two subgroups. The first subgroup included residents in their final years of training as well as board-certified neurosurgeons with fewer than five years of experience in pediatric neurosurgery; these surgeons were defined as junior surgeons (JS). The second subgroup included board-certified pediatric neurosurgeons or specialists with more than five years of experience in pediatric neurosurgery, who were defined as senior surgeons (SS).

### Statistical analysis

The statistical analysis was performed using R (version 4.3.3 and R Studio version 2024.04.0, Posit Software, Boston, MA, USA) and SPSS (version 29.0, IBM Corp., Armonk, NY, USA). The analysis included descriptive statistics, bivariate comparisons using the Fisher’s exact test or the chi-squared test as appropriate, uni- and multivariable logistic regression models adjusted for age and gender to identify independent predictors for each complication. Results were expressed as odds ratios (OR) with 95% confidence intervals (CI). Results with p < 0.05 were considered statistically significant. Time-to-event was analysed using the Kaplan–Meier method. Mean revision-free survival time with 95% confidence intervals were estimated for each group. Survival curves were compared using the log-rank (Mantel–Cox) test.

## Results

### Population characteristics

Our study included 101 patients (39 females and 62 males, mean age 7.1 ± 5.7 years); 22 patients (22.0%) underwent the BS technique, and 79 patients (78.0%) the DT technique. In 81 patients (80.0%), the EVD was positioned in frontal position, in 18 (18.0%) in occipital position, and in 2 (2.0%) in both locations. Overall, 25 drain placements (25.0%) were performed on the left side, 68 (67.0%) on the right side, and 8 (7.9%) on both sides; 90 procedures (89.0%) were performed in the operating room and 11 (11.0%) in the ICU. 28 EVD placements (28.0%) were conducted by JS and 73 (72.0%) by SS. In the entire population, the following complications were observed: 22 revisions (22.0%), 11 multiple insertion attempts (11.0%), 10 malpositionings (9.9%), and 4 bleedings (4.0%). In the subgroup of 87 patients with CSF data and no history of infections (32 females and 55 males, mean age 7.3 ± 5.6 years), the BS technique was used in 19 patients (22%), and the DT technique in 68 (78%). The EVD was inserted frontal in 69 patients (79%), occipital in 16 (18%), and in 2 (2%) in both locations. The EVD was placed on the right side in 61 patients (70%), on the left in 18 (21%), and bilaterally in 8 (9%). Most insertions were performed in the OP (n = 76, 87% vs. ICU n = 11, 13%). JS performed 25 insertions (29%), and SS 62 (71%). CSF infection occurred in 13 patients (15%), while 74 (85%) showed no evidence of infection. The baseline data are summarized in Table 1.

**Table 1:** Patient characteristics at baseline

Full cohort (n = 101)	n (%) or Mean (± SD)
Age	7.12 (± 5.73) years
Sex (female, male)	39 (39%), 62 (61%)
Technique (BS, DT)	22 (22%), 79 (78%)
Site (frontal, occipital, both)	81 (80%), 18 (18%), 2 (2%)
Side (left, right, both)	25 (25%), 68 (67%), 8 (7.9%)
Setting (OP, ICU)	90 (89%), 11 (11%)
Surgeon (JS, SS)	28 (28%), 73 (72%)
MIA (no, yes)	90 (89%), 11 (11%)
Malposition (no, yes)	91 (90%), 10 (9.9%)
Bleeding (no, yes)	97 (96%), 4 (4%)
Revision (no, yes)	79 (78%), 22 (22%)
CSF subgroup (n = 87)	**n (%) or Mean (± SD)**
Age	7.26 (± 5.56) years
Sex (female, male)	32 (37%), 55 (63%)
Technique (BS, DT)	19 (22%), 68 (78%)
Site (frontal, occipital, both)	69 (79%), 16 (18%), 2 (2.3%)
Side (left, right, both)	18 (21%), 61 (70%), 8 (9.2%)
Setting (OP, ICU)	76 (87%), 11 (13%)
Surgeon (JS, SS)	25 (29%), 62 (71%)
CSF infection (no, yes)	74 (85%), 13 (15%)

Values are given as the number of patients (%) or mean (± SD)

### Impact of demographics and catheter location

Age and sex did not significantly affect the risk of multiple punctures (*p* = 0.962 and p = 0.196), malpositioning (*p* = 0.726 and 0.309), bleeding (*p* = 0.726 and *p* = 0.639), or revision (*p* = 0.563 and *p* = 1.000). Also, the site and side of catheter placement showed no significant association with complications. A non-significant trend toward higher revision rates was observed for frontal catheters (25% vs. 6% in occipital; p = 0.098). In the subgroup analysis (*n* = 87) CSF infections occurred in 10 frontal placements (14.5%), in 1 occipital placement (6.2%), and in 2 placements in both locations (100%), showing a significant difference in catheter site (*p* = 0.018). There was a non-significant trend with regard to the side of catheter insertion: no infections occurred with left-sided placements, compared to 12 (19.7%) with right-sided placements and 1 (12.5%) with bilateral placements (*p* = 0.101). Age and sex were not associated with the presence of infection (*p* = 0.841 and *p* = 0.760). These data are summarized in Table 2.

**Table 2:** Bivariate associations between patient-, procedure-, and surgeon-related predictors and early complications after EVD placement in children

Predictor	Outcome	Distribution	p-value
**Technique (BS vs. DT)**	MIA	6 (27.3%) vs 5 (6.3%)	**0.012**
	Malposition	5 (22.7%) vs 5 (6.3%)	**0.038**
	Revision	9 (40.9%) vs 13 (16.5%)	**0.020**
**Site (frontal vs. occipital)**	MIA	10 (12.3%) vs 1 (5.6%)	0.304
	Revision	20 (24.7%) vs 1 (5.6%)	0.098
**Site (frontal vs. occipital vs. both)**	Infection*	10 (14.5%) vs 1 (6.2%) vs 2 (100%)	**0.018**
**Surgeon (JS vs SS)**	MIA	7 (25.0%) vs 4 (5.5%)	**0.009**
	Revision	11 (39.3%) vs 11 (15.1%)	**0.018**
**Setting (ICU vs OP)**	Malposition	3 (27.3%) vs 7 (7.8%)	0.076
	Revision	7 (63.6%) vs 15 (16.7%)	**0.002**
	Infection*	4 (36.4%) vs 9 (11.8%)	0.056

Distribution of events is shown as absolute numbers with percentages in parentheses. *P*-values less than or equal to 0.05 are highlighted in bold. * *Analyses were performed in a subgroup of 87 patients as described in the Methods section*

### Impact of surgical techniques

**Fig. 1:**
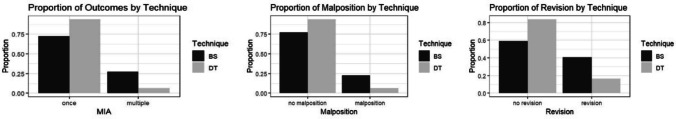
Proportions of complications (MIA, malposition, and revision) by surgical technique (BS vs. DT)

**Fig. 2:**
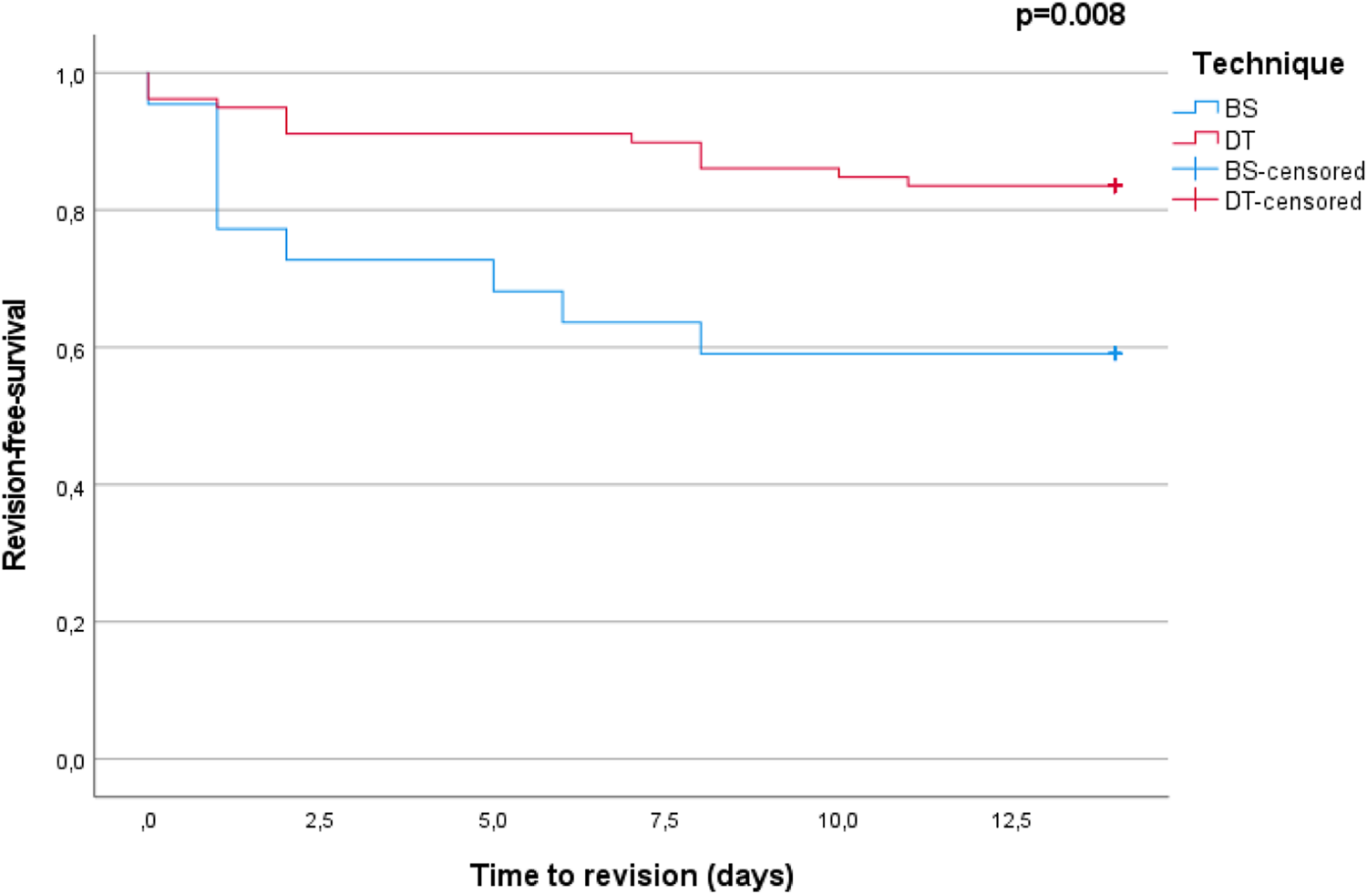
Kaplan–Meier curves demonstrating revision-free survival according to surgical technique. Patients treated with DT showed significantly higher revision-free survival compared with patients treated with BS. *The x-axis represents time to revision in days, and the y-axis represents the probability of revision-free survival. Tick marks indicate censored observations*

Of the 22 patients treated with BS, 6 (27.3%) had multiple insertion attempts (MIA) and 16 (72.7%) had a single insertion, whereas among the 79 patients treated with DT, 5 (6.3%) had MIA and 74 (93.7%) had a single insertion. According to the bivariate analysis, the incidence of MIA was significantly higher in the BS group (p = 0.012). Among patients treated with BS, 5 (22.7%) demonstrated malpositioning, compared with 17 (77.3%) who did not. Correspondingly, among the patients treated with DT, 5 (6.3%) demonstrated malpositioning and 74 (93.7%) did not (p = 0.038). In the group of patients treated with BS, 9 (40.9%) experienced revision and 13 (59.1%) did not, whereas in the group treated with DT, 13 (16.5%) experienced revision and 66 (83.5%) did not. Revision was significantly more frequently required by patients treated with BS (p = 0.020) (Fig. 1). Mean revision-free survival time was 12.44 days (95% CI 11.59–13.30) in the DT group and 9.41 days (95% CI 7.00–11.82) in the BS group. Kaplan–Meier analysis demonstrated significantly improved revision-free survival in the DT group compared with the BS group (*p* = 0.008). (Fig. 2). Among patients who underwent revision surgery, the mean time-to-revision was 2.78 days (95% CI 0.94–4.62) in the BS group and 4.54 days (95% CI 2.28–6.80) in the DT group. The difference between groups was not statistically significant (*p* = 0.157). Of the patients treated with BS, 2 (9.1%) experienced bleeding and 20 (90.9%) did not, whereas among the patients treated with DT, 2 (2.5%) experienced bleeding and 77 (97.5%) did not. Bleeding occurred more frequently after BS placement, but the difference did not reach statistical significance (*p* = 0.206). Although in the subgroup analysis (*n* = 87) the incidence of CSF infection was higher in patients treated with BS (n = 5, 26.5%) than in patients treated with DT (n = 8, 11.8%), this difference was not statistically significant (*p* = 0.147).

### Impact of surgeon experience

**Fig. 3:**
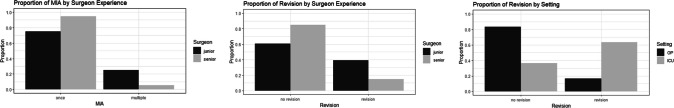
Proportions of complications by surgeon experience (**a**-**d**) and operative setting (**d**-**e**)

**Fig. 4:**
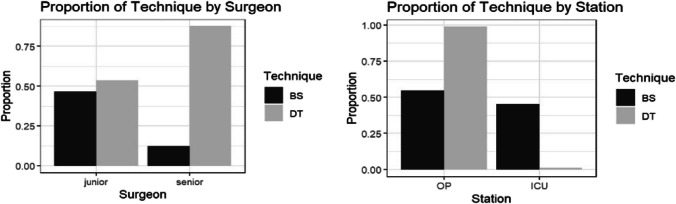
Proportions of surgical techniques by surgeon experience (**a**,**b**) and operative setting (**c**,**d**)

MIA were required after 7 procedures (25.0%) performed by JS compared to 4 (5.5%) performed by SS (p = 0.009). Revision was required in 11 procedures (39.3%) performed by JS, compared to 11 (15.1%) performed by SS (p = 0.018) (Fig. 3). Although malpositioning (17.9% vs. 6.8%) and bleeding (7.1% vs. 2.7%) occurred more frequently after JS compared to SS procedures, the difference was not significant (p = 0.199 and p = 0.307). JS used the BS technique in 13 cases (46.4%), whereas SS used BS in only 9 cases (12.3%). Conversely, the DT technique was employed in 65 EVD placements (87.7%) performed by SS compared to 15 (53.6%) performed by JS. This difference was statistically significant (p < 0.001) (Fig. 4). In the subgroup analysis infection rates were higher after insertions by JS (n = 5, 20.0%) compared to SS (n = 8, 12.9%), but this difference was not statistically significant (p = 0.508). These data are summarized in Table 2.

### Impact of operative setting

Revision was required after 7 insertions (63.6%) in the ICU compared to 15 (16.7%) in the OP, which represents a statistically significant difference (*p* = 0.002). malpositioning occurred in 3 insertions (27.3%) in the ICU vs. 7 (7.8%) in the OP, which represents a trend but does not reach statistical significance (p = 0.076). the incidence of MIA and bleeding did not differ significantly between the two groups (*p* = 0.342 and p = 0.374). the BS technique was predominantly used in the ICU compared to the OP (90.0% vs. 19.6%), whereas the DT technique was almost exclusively applied in the OP (86.7% vs. 9.1%). this association was statistically significant (*p* < 0.0001) (Fig. 4). CSF infection occurred after 4 insertions (36.4%) in the ICU, compared to 9 (11.8%) in the OP. although this difference did not reach statistical significance, the trend was notable (*p* = 0.056). these data are summarized in Table 2.

## Multivariable models

**Fig. 5:**
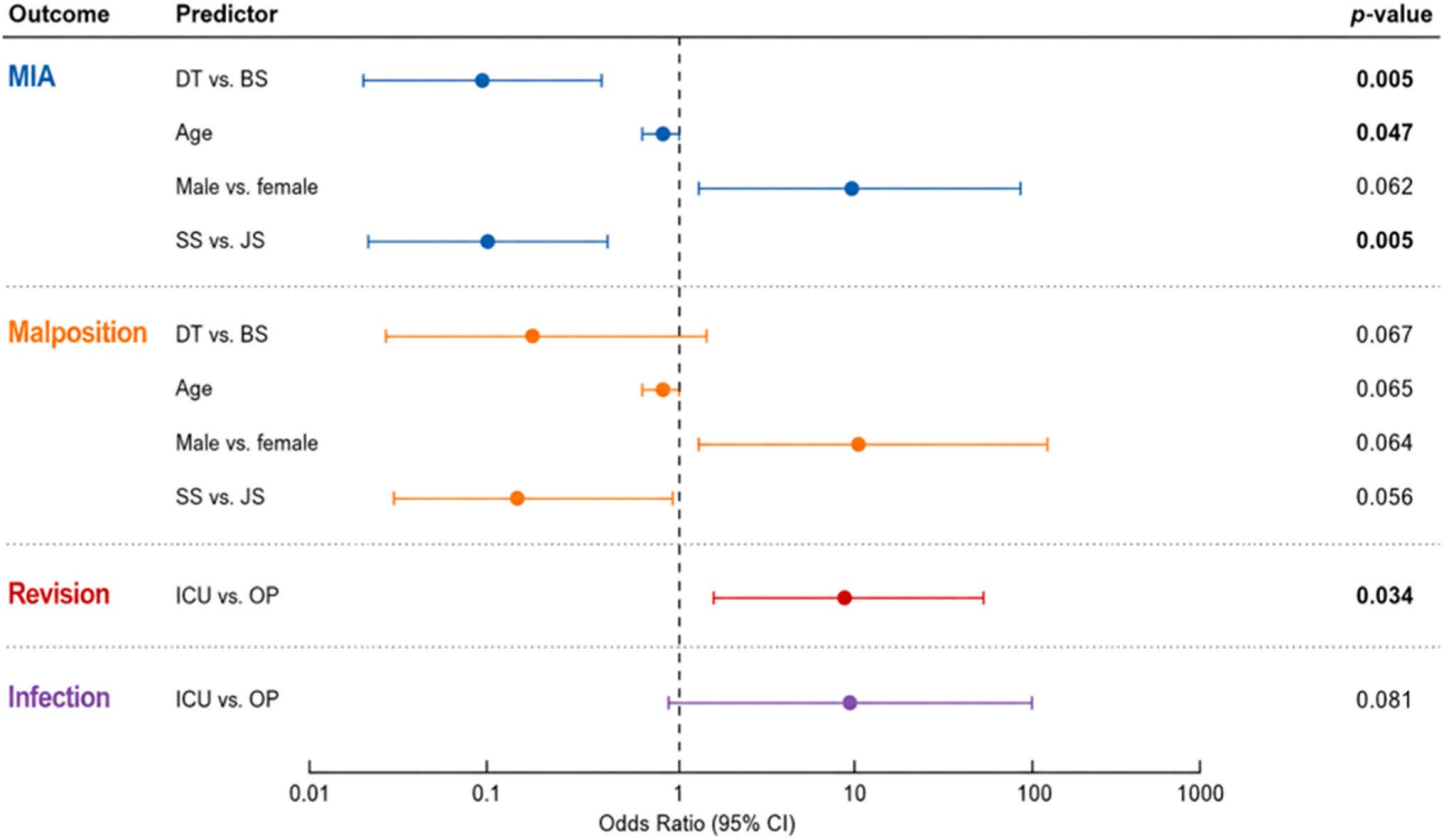
Forest plot of multivariate logistic regression analyses for predictors of early complications following EVD placement in children. odds ratios (OR) with 95% confidence intervals (CI) are shown for multiple insertion attempts (blue), malposition (orange), revision (red), and CSF infection (purple). O*nly trends and significant predictors are shown in the figure*. *Significant predictors* (*p* < *0.05*) *are highlighted in bold*. *Analyses were performed in a subgroup of 87 patients as described in the methods section

Multivariable logistic regression analyses were conducted to identify independent predictors, with each complication evaluated separately as a potential outcome. For MIA, the DT technique (vs. BS) was strongly protective (OR 0.044, 95% CI 0.004–0.339, p = 0.005), as was SS experience (OR 0.066, 95% CI 0.008–0.381, p = 0.005). Older age was also associated with fewer MIA (OR 0.820, 95% CI 0.653–0.980, p = 0.047). Male sex showed a trend toward a higher risk (OR 9.580, 95% CI 1.177–141.558, p = 0.062). For malpositioning, DT compared to BS showed a strong but non-significant protective effect (OR 0.122, 95% CI 0.011–1.222, p = 0.067). Younger age and male sex tended to be associated with higher risk (OR 0.810, 95% CI 0.625–0.987, p = 0.065 and OR 12.928, 95% CI 1.350–350.039, p = 0.064). The variable SS showed a borderline protective effect (OR 0.144, 95% CI 0.016–0.982, p = 0.056). For revision, placement in the ICU was the only independent predictor and was associated with a significantly increased risk (OR 8.966, 95% CI 1.287–80.489, p = 0.034). For bleeding, none of the independent predictors reached statistical significance. In the multivariable logistic regression analysis of the CSF infection subgroups (N = 87), none of the investigated predictors reached statistical significance. These data are summarized in Table 3 and Fig. 5.

**Table 3:** Multivariate logistic regression models assessing predictors of early complications after EVD placement in children

Outcome	Predictor	OR	95% CI	p-value
**MIA**	DT (reference BS)	0.044	0.004–0.339	**0.005**
	Age (per year)	0.820	0.653–0.980	**0.047**
	Male (reference female)	9.580	1.177–141.558	0.062
	SS (reference JS)	0.066	0.008–0.381	**0.005**
**Malposition**	DT (reference BS)	0.122	0.011–1.222	0.067
	Age (per year)	0.810	0.625–0.987	0.065
	Male (reference female)	12.928	1.350–350.039	0.064
	SS (reference JS)	0.144	0.016–0.982	0.056
**Revision**	ICU (reference OP)	8.966	1.287–80.489	**0.034**
Infection*	ICU (reference OP)	9.960	0.885–177.640	0.081

OR are reported with 95% CI. Models additionally adjusted for age and sex. P-values less than or equal to 0.05 are highlighted in bold. * *Analyses were performed in a subgroup of 87 patients as described in the Methods section*

## Discussion

Our study provides a comparative analysis of the benefits and drawbacks of the BS and DT techniques as well as of the impact of surgical experience and the operative setting on early complications after EVD placement in children. We found that the BS technique was associated with significantly higher rates of MIA, malpositioning, and revision than the DT technique, while bleeding did not differ between the two techniques. Surgeon experience also played an important role, as placements performed by JS showed higher complication rates than those performed by SS. Catheter site influenced CSF infection, which was particularly frequent in frontal placements. However, this association did not persist after multivariate adjustment, potentially reflecting the limited sample size and confounding by operative setting. A trend toward a higher infection risk was also observed in ICU procedures. In a multivariate analysis, BS, JS, and younger patient age were found to be independent predictors of MIA, whereas placement in the ICU was the only independent predictor of revision.

The development of hydrocephalus involves mostly pathological processes that affect ventricular outflow, subarachnoid space function, or cerebral venous compliance [1]. Intraventricular hemorrhage (IHV) plays a major role in the development of hydrocephalus, constituting its most severe complication [15, 16]. Tumor-associated hydrocephalus occurs in 50–90% of brain tumors in children due to the blockage of CSF flow at different anatomical levels by midline or paramidline tumors [17, 18]. Pediatric posterior fossa brain tumors also commonly present with hydrocephalus [3, 19–21]. EVD insertion represents one of the temporary treatment modalities in neurosurgical practice and plays a key role in CSF drainage and parallel intracranial pressure (ICP) monitoring, particularly in patients with impaired consciousness. EVD insertion also serves to externalize CSF contaminated with protein or bacteria from the ventricular system [3, 16, 22, 23]. Despite its inherent risks of hemorrhage, infection, and misplacement, EVD placement is often considered a simple procedure [9]. Over time, many changes to EVD insertion techniques have been suggested to reduce the risk of these complications [7]. At our clinic, both techniques are routinely employed in daily practice; however, given the favorable outcomes reported in adults [10, 11], the BS technique is usually preferred to the DT technique.

In our pediatric population, the incidence of CSF infection was higher in patients treated with BS than in patients treated with DT. However, this difference was not statistically significant. In contrast, Jensen et al. (2016) reported higher infection rates after DT, although the difference did not reach statistical significance [12]. The difference to the adult population becomes even more evident when considering data from patients over the age of 18 at our clinic, where infection rates were significantly higher after DT [10, 11]. In contrast to other authors [24, 25], the site of the catheter was significantly associated with infection. However, this association did not persist after adjusting for other factors in multivariate models.

Our study shows a significant conceptual difference in surgical complications between adult and pediatric populations. Whereas EVD insertion using a BS appears advantageous in adults, this technique is less favorable in children. The significantly higher rates of MIA, malpositioning, and revision associated with the BS technique in univariate analyses, together with the strong protective effect of the DT procedure on MIA in multivariate models, support the conclusion that DT is the preferable technique in the pediatric setting. Even when accounting for the surgical setting, substantial differences emerge between adult and pediatric populations. In our cohort, revisions were significantly more frequently required after ICU insertions, and there was a trend toward higher infection rates compared to OP procedures, whereas several adult studies have reported the opposite pattern [7, 9].

Regarding the methods of classifying surgical experience, some authors have used a similar procedure. This procedure shows that the accuracy of bolt EVD insertions depends on neurosurgical experience, and the association persists throughout the different stages of training [26]. Conversely, other authors have reported that the risk of complications and the accuracy of drainage placement are not influenced by surgeon experience [6, 27]. Interestingly, our data show that JS performed BS insertions more often, whereas SS predominantly used DT, which points out a significant association between surgeon experience and the chosen surgical technique. This distribution may represent a confounding factor, but it also underscores the potential relevance of the method itself rather than surgeon experience alone. Accordingly, we performed multivariate analyses considering each complication individually. These analyses revealed that both surgical technique and surgeon experience are independent predictive factors for specific outcomes. The BS technique was used significantly more often in the ICU than in the OP, and the DT technique was almost exclusively used in the OP. However, in the multivariate analysis of revision as a specific outcome, EVD placement in the ICU setting emerged as the only independent predictive factor, associated with nearly nine times higher odds of revision.

The overall rate of EVD malpositioning in our cohort was consistent with that described in the literature [28–32]. These high rates are particularly relevant in the pediatric setting, where a higher level of accuracy is expected. They underscore the need to reflect on the role of technological adjuncts in the implantation process. Mobile navigation instruments and image guidance may enable drain accuracy, reduce the drain failure rate, and enhance the safety and efficiency of EVD insertion [30, 33–35], especially when supported by simple tools that do not require much effort [30, 36–38].

## Limitation

Our study has several limitations. The first limitation is its single-center setting. Because of its importance, this topic deserves further investigation and validation with larger multicentric cohorts. Second, the relatively small size of the overall cohort limits the statistical power, resulting in wide confidence intervals for some predictors. Moreover, the choice of surgical technique was not randomized but determined by institutional practice, patient age, and surgeon preference. As the BS technique was more frequently employed by junior surgeons and in the ICU setting, potential confounding between the technique, surgeon experience, and the operative environment cannot be entirely excluded, despite adjustment in multivariate models. Possible further limitations of the study are the short follow-up and the lack of long-term outcome data. Finally, since all children received antibiotic-impregnated catheters in accordance with institutional standards, the observed infection rates may not be directly comparable to centers where standard catheters are still in use.

## Conclusion

In pediatric patients, EVD placement using the DT technique demonstrated greater safety, with a lower risk of MIA, malpositioning, and revision than the BS technique. Outcomes were additionally influenced by surgeon experience and the operative setting, which underscores the importance of selecting the right technique and considering the procedural context to minimize complications.

## Data Availability

Raw data will be made available on reasonable request via the corresponding authors.

## Abbreviations

CSF: Cerebrospinal fluid
EVD: External ventricular drain
BS: Boltkit screw
DT: Drain tunneling
OP: Operating room
ICU: Intensive care unit
JS: Junior surgeons
SS: Senior surgeons
SAP: Systems, Applications & Products in Data Processing
MIA: Multiple insertion attempts
CI: Confidence interval
OR: Odds ratio
SD: Standard deviation
